# Supplementary material for: Delaying cancer progression by integrating toxicity constraints in a model of adaptive therapy
Source: NPJ Syst Biol Appl. 2026 Jan 8;12:11. doi: 10.1038/s41540-025-00635-6 (PMC12820236; doi:10.1038/s41540-025-00635-6)
Supplement: Supplementary file 1 — Supplementary information [file 41540_2025_635_MOESM1_ESM.pdf]

Supplementary Information: Delaying Cancer Progression by Integrating Toxicity Constraints in a Model of Adaptive Therapy

Jana L. Gevertz, Harsh Vardhan Jain, Irina Kareva, Kathleen P. Wilkie, Joel Brown, Yitong Pepper Huang, Eduardo Sontag, Vladimir Vinogradov, Mark Davies

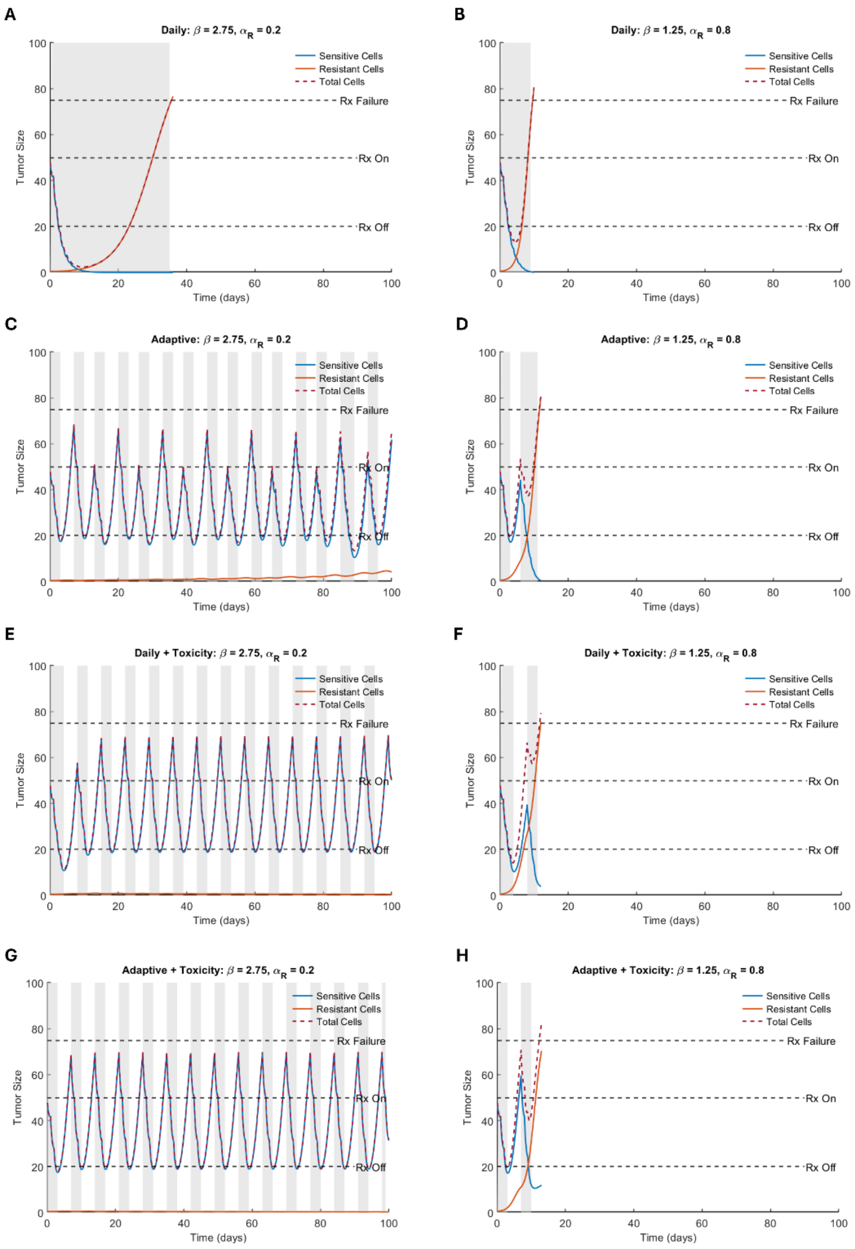

Supplementary Figure 1. The four treatment protocols and resulting model outputs. The left column shows a parameterization for which resistant cells are slow growing but have a strong competitive advantage. The right column shows a parameterization for which resistant cells are fast growing but have a weaker competitive advantage. Compared protocols are daily (A-B), adaptive (C-D), daily with toxicity feedback (E-F), and adaptive with toxicity feedback (G-H). Time periods shaded grey represent when the treatment is on.

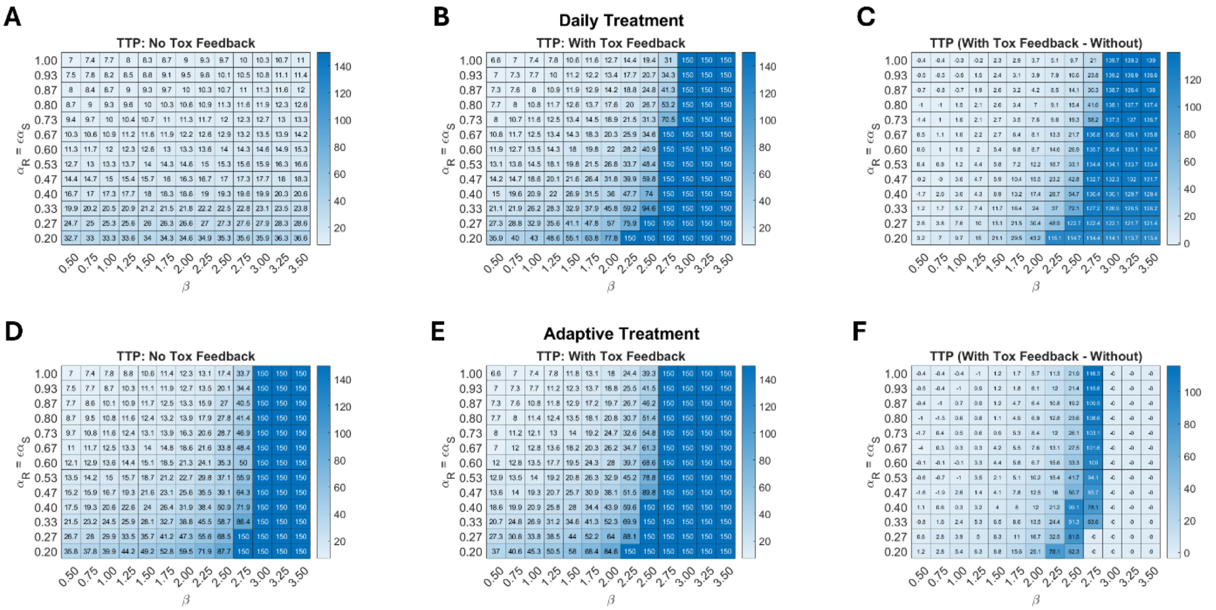

Supplementary Figure 2. Parameter sweeps over $\beta - \alpha_R$ space. TTP is shown across parameter space for (A) daily protocol, (B) daily protocol with toxicity feedback, (D) adaptive protocol, (E) adaptive protocol with toxicity feedback. A value of 150 indicates that the predicted tumor did not progress within the simulation time of 100 days. The remaining parameters are fixed at their baseline value in Table 1. The right column shows the difference in TTP comparing the protocol with toxicity feedback to the same protocol without toxicity feedback: (C) daily, (F) adaptive.

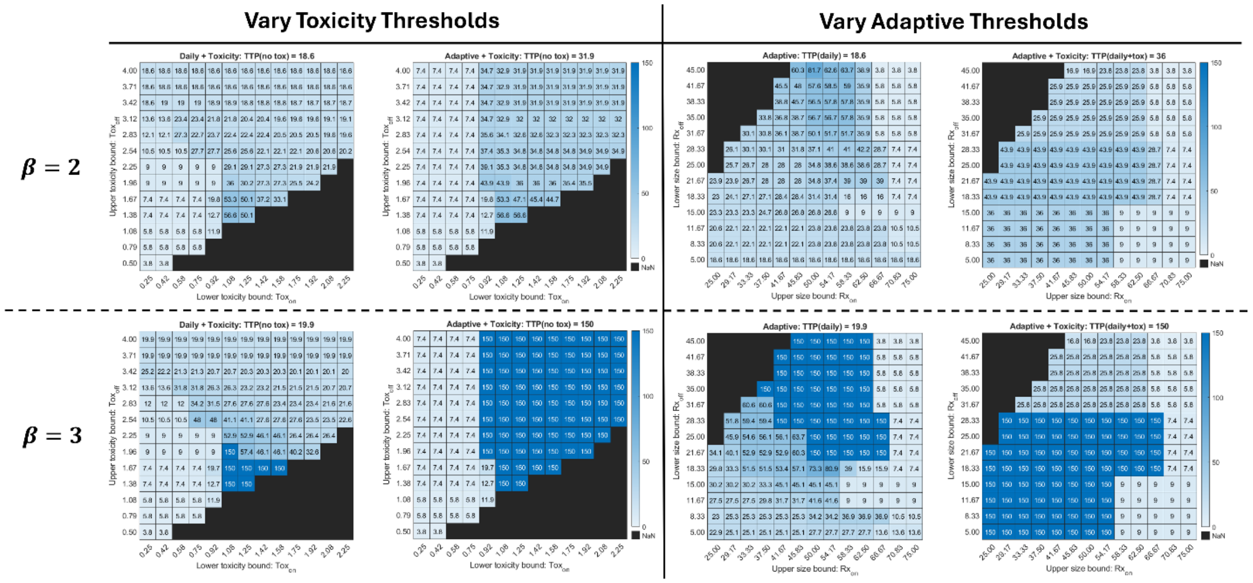

Supplementary Figure 3. Effect of β on protocol sweeps.

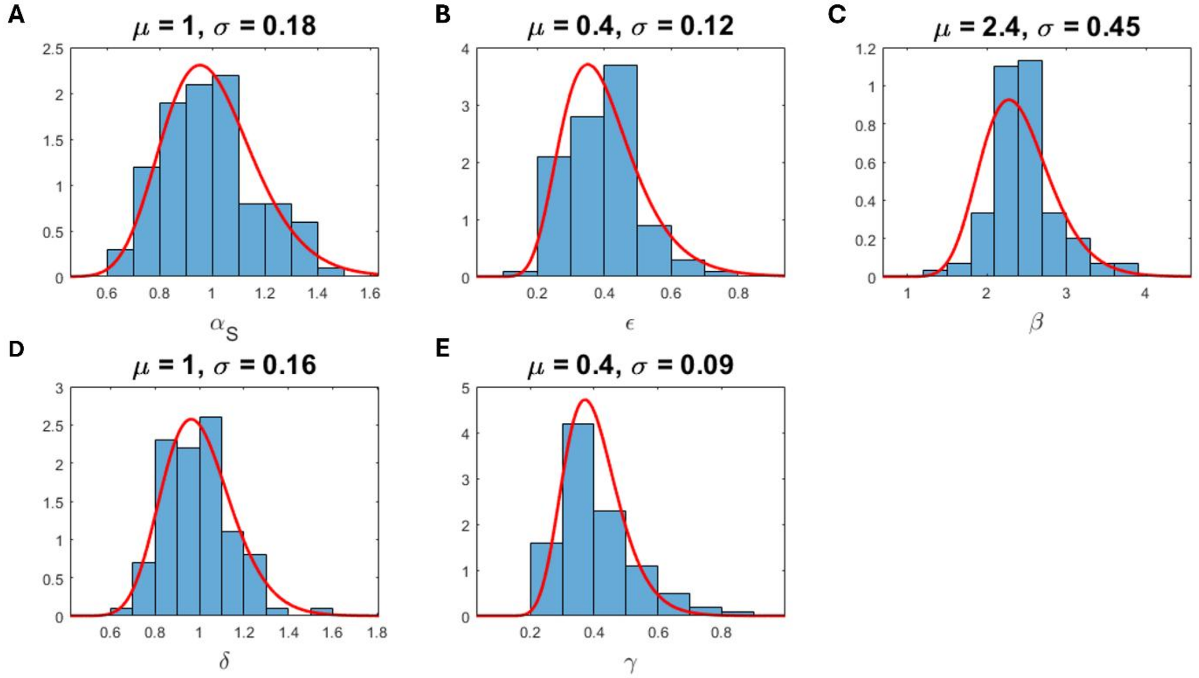

Supplementary Figure 4. Lognormal distributions defined for each parameter of the VP analysis. The parameters defining the lognormal distribution (μ and σ) for each VP parameter (α_S, ϵ, β, δ, and γ) are chosen such that the peak aligns with the baseline value reported in Table 1, and the width covers the range used in the global sensitivity analysis of Figure 3.
